# Radial laser ablation under cholangioscopic guidance for a tight intrahepatic bile duct stricture caused by primary sclerosing cholangitis

**DOI:** 10.1055/a-2743-9806

**Published:** 2025-12-08

**Authors:** Nobuhiro Hattori, Takeshi Ogura, Junichi Nakamura, Takafumi Kanadani, Hiroki Nishikawa

**Affiliations:** 1 38588 2nd Department of Internal Medicine, Osaka Medical and Pharmaceutical University Hospital, Takatsuki, Japan; 2 2nd Department of Internal Medicine, Osaka Medical and Pharmaceutical University, Osaka, Japan; 3 13010 Endoscopy Center, Osaka Medical and Pharmaceutical University Hospital, Osaka, Japan

Primary sclerosing cholangitis (PSC) is an autoimmune disease caused by fibrotic strictures of the bile ducts 1 2 . In cases with obstructive jaundice due to a bile duct stricture, treatment with biliary drainage can be considered. However, stent deployment might be difficult if the biliary stricture is long and tight. To resolve the stricture, balloon dilation is the ideal procedure, but recurrence of the stricture must be considered. In contrast, the radial incision and cutting (RIC) method has shown promising results for the treatment of stricture 3 because scar tissue can be removed. However, because of the limited space of bile ducts, standard RIC may be challenging. Laser ablation can be performed under cholangioscopic guidance, so laser ablation to remove scar tissue might be an option for treating biliary strictures. Successful treatment of a tight stricture due to PSC using radial ablation under cholangioscopic guidance is described.

An 88-year-old man with obstructive jaundice due to PSC was admitted to another hospital. Plastic stent deployment was attempted, but guidewire passage into the right hepatic bile duct failed. Therefore, he was referred to our hospital for the treatment of the obstructive jaundice. First, biliary cannulation was successfully performed, and the contrast medium was then injected. On cholangiography, the left hepatic bile duct was lightly stenosed, and the right hepatic bile duct was completely obstructed ( Fig. 1 ). Although the guidewire passage was successful, balloon catheter advancement across the stricture site failed. Therefore, laser ablation was attempted. A cholangioscope was inserted, and the stricture site was identified ( Fig. 2 ). Radial laser ablation was then attempted ( Fig. 3 ), and the bile duct distal to the stricture was visualized ( Fig. 4 ). After guidewire deployment into the right hepatic bile duct, plastic stents were successfully deployed at the left and right hepatic bile ducts without any adverse events ( Fig. 5 and Video 1 ).

**Fig. 1:**
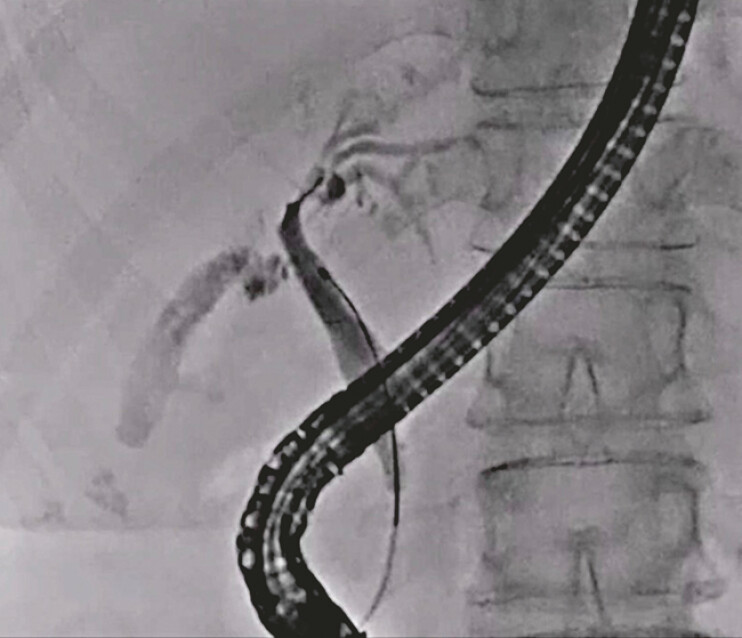
The right hepatic bile duct is completely obstructed.

**Fig. 2:**
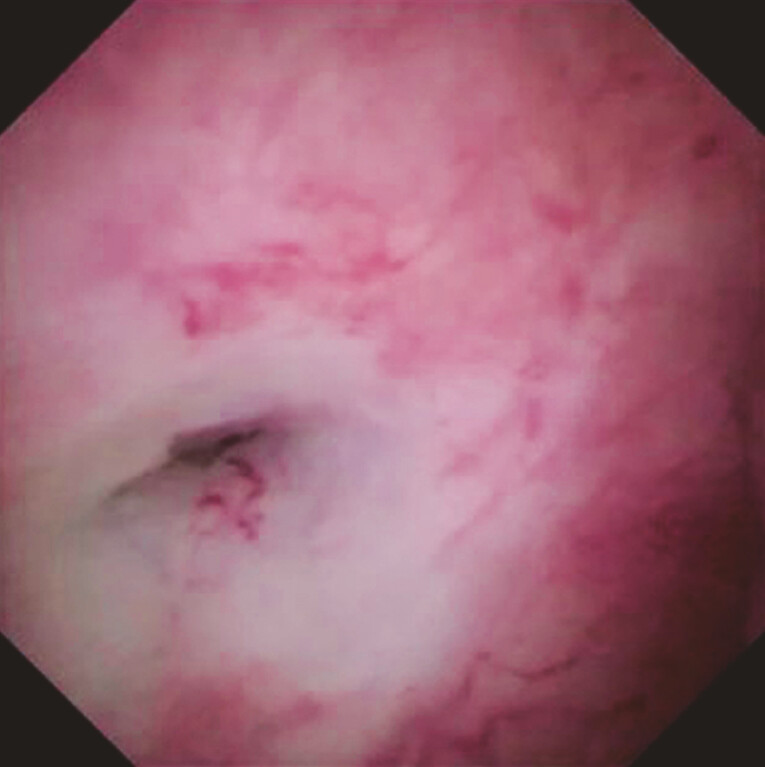
The stricture site is identified on cholangioscopic guidance.

**Fig. 3:**
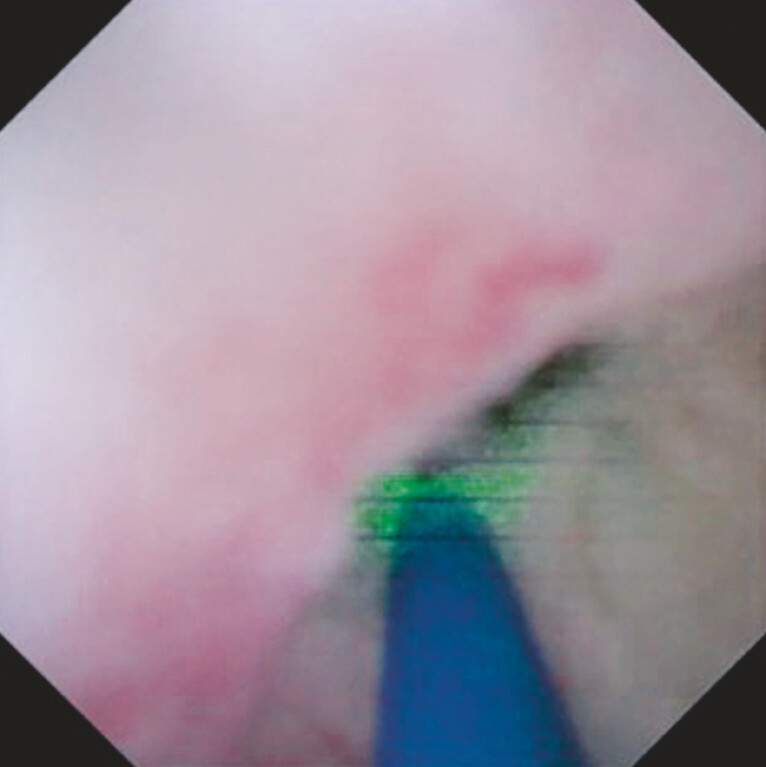
Radial laser ablation is attempted.

**Fig. 4:**
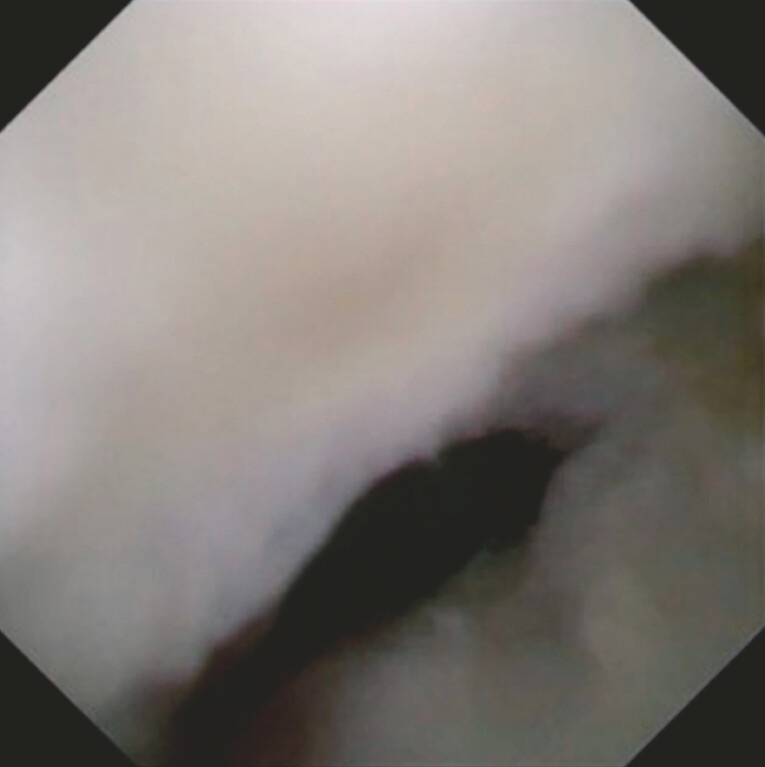
The bile duct distal to the stricture is successfully visualized.

**Fig. 5:**
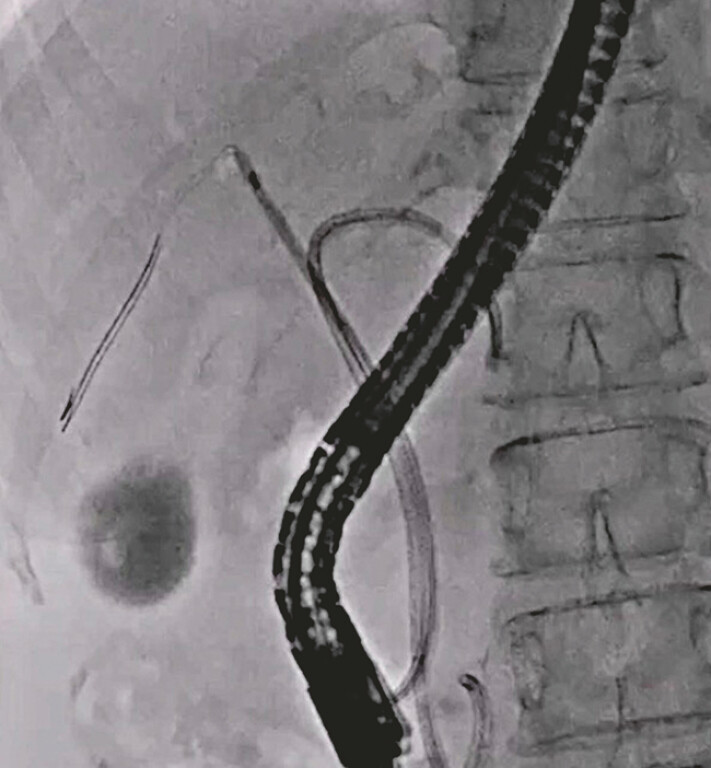
Plastic stents are successfully deployed at the left and right hepatic bile ducts.

Radial laser ablation for the tight bile duct stricture is attempted.Video 1

In conclusion, radial laser ablation might be an option for treating long and tight biliary strictures.

Endoscopy_UCTN_Code_TTT_1AR_2AG

## Notes

Endoscopy E-Videos https://eref.thieme.de/e-videos
*E-Videos* is an open access online section of the journal *Endoscopy* , reporting on interesting cases and new techniques in gastroenterological endoscopy. All papers include a high-quality video and are published with a Creative Commons CC-BY license. Endoscopy E-Videos qualify for HINARI discounts and waivers and eligibility is automatically checked during the submission process. We grant 100% waivers to articles whose corresponding authors are based in Group A countries and 50% waivers to those who are based in Group B countries as classified by Research4Life (see: https://www.research4life.org/access/eligibility/ ). This section has its own submission website at https://mc.manuscriptcentral.com/e-videos .
